# HLA-B*15:01 is associated with asymptomatic SARS-CoV-2 infection

**DOI:** 10.1101/2021.05.13.21257065

**Published:** 2021-09-10

**Authors:** Danillo G. Augusto, Tasneem Yusufali, Noah D. Peyser, Xochitl Butcher, Gregory M. Marcus, Jeffrey E. Olgin, Mark J. Pletcher, Martin Maiers, Jill A. Hollenbach

**Affiliations:** 1. Department of Neurology, University of California, San Francisco; 2. Programa de Pós-Graduação em Genética, Universidade Federal do Paraná, Curitiba, Brazil; 3. Division of Cardiology, Department of Medicine, University of California, San Francisco; 4. Department of Epidemiology and Biostatistics, University of California, San Francisco; 5. Division of General Internal Medicine, University of California, San Francisco; 6. National Marrow Donor Program, Minneapolis, MN

## Abstract

**Background.:**

Evidence has shown that a large proportion of SARS-CoV-2 infected individuals do not experience symptomatic disease. Owing to its critical role in immune response, we hypothesized that variation in the *human leukocyte antigen* (HLA) loci may underly asymptomatic infection.

**Methods.:**

We enrolled 29,947 individuals registered in the National Marrow Donor Program for whom high-resolution *HLA* genotyping data were available in a smartphone-based study designed to track COVID-19 symptoms and outcomes. Among 21,893 individuals who completed the baseline survey, our discovery (N=640) and replication (N=788) cohorts were comprised of self-identified White subjects who reported a positive test result for SARS-CoV-2. We tested for association of five *HLA* loci (*HLA-A*, *-B*, *-C*, *-DRB1*, *-DQB1*) with asymptomatic vs. symptomatic infection.

**Results.:**

HLA-B*15:01 was significantly increased in asymptomatic individuals in the discovery cohort compared to symptomatic (OR = 2.45; 95%CI 1.38–4.24, *p* = 0.0016, *p*^corr^ = 0.048), and we reproduced this association in the replication cohort (OR= 2.32; 95%CI = 1.10–4.43, *p* = 0.017). We found robust association of HLA-B*15:01 in the combined dataset (OR=2.40 95% CI = 1.54–3.64; *p* = 5.67 ×10^−5^) and observed that homozygosity of this allele increases more than eight times the chance of remaining asymptomatic after SARS-CoV-2 infection (OR = 8.58, 95%CI = 1.74–34.43, *p* = 0.003). Finally, we demonstrated the association of HLA-B*15:01 with asymptomatic SARS-Cov-2 infection is enhanced by the presence of HLA-DRB1*04:01

**Conclusion.:**

HLA-B*15:01 is strongly associated with asymptomatic infection with SARS-CoV-2 and is likely to be involved in the mechanism underlying early viral clearance.

## Introduction

Despite some inconsistent reporting of symptoms,^1^ studies have demonstrated that at least 20% of individuals infected with severe acute respiratory syndrome coronavirus 2 (SARS-CoV-2) will remain asymptomatic.^2–4^ Although global efforts have focused on understanding factors underlying severe illness in COVID-19 (coronavirus disease of 2019), the examination of asymptomatic infection provides a unique opportunity to consider early disease and immunologic features promoting rapid viral clearance. Specific focus on asymptomatic infection has the potential to further our understanding of disease pathogenesis and supports ongoing efforts toward vaccine development and the discovery of potential therapeutic targets.

It remains unclear why many individuals successfully clear infection without major complications while others develop severe disease, even in the absence of known risk factors for severe COVID-19 outcomes.^5^ However, host genetics is long-known to be implicated in differential immunological responses to infection and disease progression.^6^ Numerous studies intending to understand the genetic basis of differential outcomes in COVID-19 have been underway since nearly the start of the global pandemic, including the multicenter Host Genetics Initiative^7^. However, the vast majority of these studies have examined genetic associations with severe disease course, in primarily hospitalized cohorts.^8–10^ As a result, although most individuals infected with SARS-Cov-2 experience mild disease course or are entirely asymptomatic, very few studies have examined genetics in the context of non-hospitalized, prospective, community-based cohorts.

The *human leukocyte antigen (HLA)* region, located on chromosome 6p21, is the most polymorphic and medically important region of the human genome. Variation in *HLA* has been associated with hundreds of diseases and conditions, including infection. Infectious diseases are one of the leading causes of human mortality^11^ and are a primary selective pressure shaping the human genome.^12,13^ Among the myriad genes involved in human immune responses, HLA variants are among the strongest reported associations with viral infections. For example, *HLA* was strongly associated with the rapid progression and viral load control of HIV (human immunodeficiency virus),^14^ hepatitis B, hepatitis C, and other infectious diseases.^15^ Notably, *HLA* class I and class II alleles have also been associated with the severe acute respiratory syndrome caused by SARS-CoV.^16–21^

A series of in silico analyses have pointed to HLA as relevant molecules for SARS-CoV-2 risk and essential targets for vaccine development.^22–25^ For example, HLA-B*46:01 has low predicted binding of peptides for SARS-CoV-2, suggesting that individuals expressing this molecule may be more vulnerable to COVID-19,^24^ which corroborates previous results showing HLA-B*46:01 association with SARS risk.^18^ In contrast, HLA-B*15:03 was predicted to protect against COVID-19 with the greatest ability to present highly conserved SARS-CoV-2 peptides to T cells.^24^ More recently, it was demonstrated that while there is some overlap, many SARS-CoV-2 epitopes for CD8 T cells are *HLA* specific.^24^ To date, very few studies have directly examined HLA associations with infection, with mixed and inconclusive results in relatively small cohorts.^26–28^ Larger studies that relied on genome-wide data to impute *HLA* failed to find robust associations with disease.^8,29^ However, these studies focused primarily on hospitalized patients with severe disease course.

Because of its pivotal role in the immune response, understanding the impact of *HLA* variation in disease promises to provide meaningful insights relevant to understanding the immunopathogenesis of COVID-19, while informing vaccine development and potential immunotherapies. Here, we present the largest study to-date directly examining *HLA* variation in the context of primarily mild disease. We invited volunteer bone marrow donors for whom high-resolution *HLA* genotyping data were already available to participate in the COVID-19 Citizen Science Study, a smartphone-based study designed to track COVID-19 symptoms and outcomes, including self-reported positive tests for SARS-CoV-2 infection, to develop a prospective cohort currently numbering nearly 30,000 individuals. Our early results provide strong support for the role of HLA class I in viral clearance leading to asymptomatic infection among persons with SARS-CoV-2 infection and provide an important framework for additional studies aimed at revealing the immunological and genetic basis for recovery from SARS-CoV-2 infection.

## Methods

### Data collection

This study was conducted in accordance with the ethical standards of the UCSF Human Research Protection Program Institutional Review under approval #20–30850 and informed consent was obtained from all individual participants involved in the study. Subjects were volunteer bone marrow donors with valid email addresses on file with the National Marrow Donor Program (NMDP) who were invited to participate in the study through an email outreach campaign that began in July 2020. All subjects have within the NMDP database a pre-existing record for high-resolution *HLA* genotyping, typically for five loci (*HLA-A, -B, -C, -DRB1,* and - *DQB1*).^30^ Participants who opt in to the study are required to download a smartphone app and participate in the COVID-19 Citizen Science Study (launched using the Eureka Digital Research Platform, https://eureka.app.link/covid19) or, as of January 2021, participate via website (https://covid19.eurekaplatform.org/). Once enrolled, participants are asked to complete an initial 10- to 15-minute survey about baseline demographics, their health history and daily habits. Follow-up daily questions specific to symptoms, weekly questions regarding testing and monthly questions regarding hospitalization for COVID-19, are delivered by push notification or text message on an ongoing basis and require five to 15 minutes per week. As of April 30, 2021, we enrolled 29,947, of whom 21,893 have completed their baseline survey.

**Figure 1.**
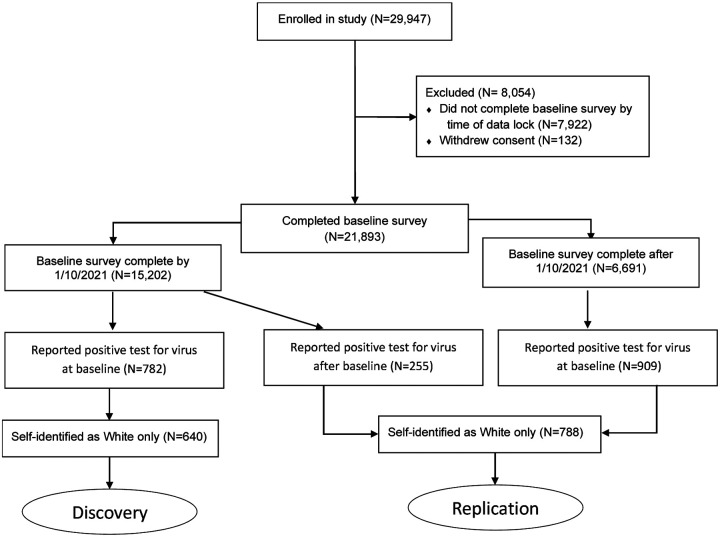
Study inclusion criteria

Within the mobile application, survey respondents are asked during their initial baseline survey whether they have ever been tested for active infection and report the result (positive, negative, do not know) and the approximate number of weeks since the test. Thereafter, each week respondents are asked whether they were tested in the prior week, and to report the result. We considered anybody reporting a positive test for active infection as having been infected with SARS-CoV-2. Our discovery cohort consisted of individuals reporting a positive test for virus at baseline at the point that we had collected baseline data for the first 15,000 respondents; we achieved this threshold on January 10, 2021. Respondents reporting a positive test for the virus after baseline (i.e., on a weekly survey) or who completed their baseline survey after January 10, 2021, together formed our replication cohort. We restricted the analysis to individuals who had self-identified as “White” only due to insufficient numbers for analysis in other groups, allowing analysis of 640 individuals in our discovery cohort and 788 individuals in our replication cohort. Inclusion criteria are detailed in Figure 1.

Symptoms are self-reported at baseline and using the daily surveys. Within the baseline survey, respondents are asked to report whether they had any of a list of symptoms (Table S1) for three days or longer at any time since February of 2020. These same symptoms are queried in each daily survey, where respondents are asked whether they experienced each symptom within the previous 24 hours. Among those individuals, we considered those who reported having had a positive test for active virus at baseline, with a time since the test of longer than two weeks or who did not specify test dates, and who reported “None of the above” for all symptoms in the baseline survey, as “asymptomatic.” We also considered daily symptom reports for the two weeks after baseline for respondents who reported a positive test for active infection at baseline as having occurred within the prior two weeks. In these cases, we considered individuals asymptomatic if in addition to reporting no symptoms at baseline, they did not report any single symptom two or more times within this time period. For individuals who did not report a positive test for active infection at baseline, but subsequently reported a positive test on a weekly survey, we used the same criteria considering daily symptom reports for the period two weeks prior and two weeks after the positive test report.

### HLA association analysis

We examined the association of five *HLA* loci (*HLA-A, -B, -C, -DRB1, -DQB1*) with asymptomatic vs. symptomatic infection. Initial testing for *HLA* associations was performed using the R package BIGDAWG,^31^ which handles multiallelic *HLA* data to test for association at the haplotype, locus, allele, and amino acid levels. We employed a generalized linear model using ‘glm’ in the R base package to consider relevant covariates, including any reported comorbidities, sex, and age, for alleles initially found to be associated with asymptomatic infection after correction for multiple testing. We corrected p-values using the Bonferroni method^32^ for the number of alleles tested at *HLA-A*, *-B*, and *-DRB1*, which allows for the strong linkage disequilibrium between some loci tested.

### Role of the funding source

The funders of the study had no role in study design, data collection, data analysis, data interpretation, or writing of the report.

## Results

### Study population characteristics

Our study population was overwhelmingly comprised of individuals with mild disease, with only two individuals reporting hospitalization for COVID-19. The discovery cohort consisted of 640 individuals who reported a positive test for active SARS-CoV-2 infection at baseline and self-identified as White. Among these respondents, 90 (14.1%) reported having remained asymptomatic for at least two weeks after a positive test for virus. Among the 788 individuals who reported a positive test result for SARS-CoV-2 active infection and self-reported as White who comprised our replication cohort, 46 (5.8%) were asymptomatic according to our study criteria.

Median age was increased (*p* < 0.001) in asymptomatic compared to symptomatic individuals in each cohort (discovery asymptomatic=44, symptomatic=37; replication asymptomatic =38, symptomatic=31). Likewise, median age in our discovery cohort overall (38) was higher than our replication cohort (31; p<0.001). Finally, while not statistically significant, there was a trend toward increased asymptomatic infection in males (*p*=0.07) in our discovery cohort. Basic demographics for all subjects are given in Table 1.

We also collected data on several diseases and conditions that might impact COVID-19 disease course. Among all individuals who reported a positive test for virus across both cohorts (N=1,428), 67% reported no known COVID-19-associated comorbidities. We did not observe a significant difference in any reported comorbidity between the discovery and replication cohorts. The full list of reported diseases and conditions is given Table S2

### HLA-B*15:01 is associated with asymptomatic SARS-CoV-2 infection

We aimed to identify whether HLA variation impacts the likelihood that an individual will remain asymptomatic after SARS-CoV-2 infection. We analyzed high-resolution genotyping for five highly polymorphic *HLA* class I and class II genes (*HLA-A*, *HLA-B*, *HLA-C*, *HLA-DRB1*, *HLA-DQB1*) in each study cohort. Data analysis included the first two fields of the allele name as described in the HLA nomenclature, representing the complete molecule at polypeptide sequence resolution.

In the discovery cohort, we found the allele HLA-B*15:01 significantly overrepresented in asymptomatic individuals relative to symptomatic individuals (*f*=0.11 vs. 0.047; OR=2.52; 95%CI 1.39–4.42, *p* = 0.00057, *p*^corr^ = 0.017, Table 2). No other *HLA* allele at any locus was found to be significantly associated after correction for multiple comparisons. Allelic frequencies for all loci are given in Tables S3–S7.

To adjust for the effect of comorbid conditions, as well as sex and age differences in asymptomatic vs. symptomatic patients, we fitted a series of regression models but did not find any impact of patient-reported comorbidities on the likelihood of asymptomatic disease. Thus, our final model adjusted only for age and sex, which again showed a significant association of HLA-B*15:01 with asymptomatic infection after adjustment for these variables (OR = 2.45; 95%CI 1.38–4.24, *p* = 0.0016, *p*^corr^ = 0.048, Table 2).

We reproduced the association of HLA-B*15:01 with asymptomatic infection (Table 2, sex and age adjusted) in the replication cohort and found a remarkably consistent effect size for this allele (OR= 2.32; 95%CI = 1.10–4.43, *p* = 0.017). Given the consistency between the discovery and replication cohort observations, we examined the combined dataset for an overall association of HLA-B*15:01 with asymptomatic infection (OR=2.40 95% CI = 1.54–3.64; *p* = 5.67 ×10^−5^). Finally, we observed a strong additive effect for the associated genotype in the combined dataset. Individuals who carry two copies of HLA-B*15:01 are more than eight times more likely to remain asymptomatic than individuals carrying other genotypes (OR = 8.58, 95%CI = 1.74–34.43, *p* = 0.003). Overall, one in five individuals (20%) who remained asymptomatic after infection carried HLA-B*15:01, compared to 9% among patients reporting symptoms.

### HLA-B*15:01 association with asymptomatic SARS-Cov-2 infection is enhanced by the presence of HLA-DRB1*04:01

To understand whether additional *HLA* alleles might interact with HLA-B*15:01 in asymptomatic infection, we tested all pairwise two-locus haplotypes containing *HLA-B* in the combined dataset. Overall, haplotypic associations for *HLA-B~HLA-DRB1* and *HLA-A~HLA-B* were found to be significant at *p*=0.01. Examining specific allelic haplotypes, these associations were driven by two HLA-B*15:01 haplotypes: HLA-B*15:01~HLA-DRB1*04:01, and HLA-A*02:01~HLA-B*15:01 (Tables S8–9).

After adjusting for sex and age, only the combination of HLA-B*15:01 and HLA-DRB1*04:01 remained significant after correction for multiple comparisons (*p* = 3 × 10^−4^, *p*^corr^ = 0.01). We found an odds ratio for this combination (OR=3.17, 95% CI=1.65–5.80) that exceeds that for HLA-B*15:01 alone, suggesting that while not significantly associated with the asymptomatic infection on its own, the class II allele HLA-DRB1*04:01 enhances the effect of HLA-B*15:01.

## Discussion

Leveraging big data and mobile technology in this crowd-sourced study, we reveal important insight into the immunogenetic underpinnings of asymptomatic SARS-CoV-2 infection. Our innovative use of a mobile application and a pre-existing database for medical research allowed us to screen nearly 30,000 individuals previously genotyped for *HLA* for viral infection and disease course.

Among participants reporting a positive test result for SARS-CoV-2, HLA-B*15:01 is significantly associated with asymptomatic infection. We observed that individuals carrying this relatively common allele are more than twice as likely to remain asymptomatic after SARS-CoV-2 infection than those who do not, and an astonishing effect for HLA-B*15:01 homozygosity increasing more than eight times the chance of remaining asymptomatic. This observation suggests important features of early infection with SARS-CoV-2.

Although our data do not point to a direct mechanism, compelling evidence indicates that B*15:01 is predicted to bind a specific SARS-CoV-2 epitope, WTAGAAAYY, with high affinity.^33–38^ This epitope has been considered a natural candidate for SARS-Cov-2 vaccine studies^39,40^ as it is considered to be highly immunogenic and predicted to elicit a strong CD8+ T cell responses.^41–44^ The dominant recognition and consequent eliciting of immune responses by only a relatively small number of epitopes–immunodominance – is determined by multiple factors, including the binding affinity of the peptide for the HLA molecule.^45^ We speculate that the high affinity of HLA-B*15:01 for binding WTAGAAAYY or another unknown viral peptide confers strong CD8+ T cell responses against SARS-CoV-2 infected cells, leading to better early viral control and thus resulting in asymptomatic course of infection. An alternative explanation for our results is that HLA-B*15:01 carriers previously infected by other common coronaviruses have pre-existing cross-reactive T-cells with high affinity for a specific epitope conferred by this HLA molecule. While the current literature is mixed regarding cross-reactive CD8+ T cells specific to SARS-CoV-2, this might be explained by HLA specificity.^46,47^ The fact that asymptomatic individuals in our study were less likely than symptomatic subjects to report a positive test for antibodies against SARS-CoV-2 (Table S10) suggests a strong early anti-viral response and may corroborate the critical role of CD8+ T cytotoxicity mediated by HLA class I in eliminating SARS-CoV-2 infected cells.

A study from England has recently reported a suggestive association of HLA-DRB1*04:01 with asymptomatic infection.^27^ While our results did not corroborate this association for HLA-DRB1*04:01 alone, we did find that this allele enhanced the effect of HLA-B*15:01 when the pair were in combination. We note that this is the *HLA-DRB1* allele most commonly associated with HLA-B*15:01 in individuals in the U.S. who self-identify as White, and thus it is difficult to differentiate a real effect from one related to linkage disequilibrium between these loci. We speculate that the study by Langton et al.^27^ may have been detecting the same HLA-B*15:01 association observed here, but due to the high number of alleles commonly observed at *HLA-B*, that study may have been insufficiently powered to detect the association.

A key limitation of this study is that all testing results and symptoms are self-reported. We recognize that this may result in some margin of error in our results. However, we have previously validated this approach by verifying test results in a subset of the participants.^48^ Additionally, we note that we find a remarkably consistent genetic association across the study, pointing to a true biological feature.

Likewise, some key differences were observed between the discovery and replication phase of our study. For example, differences in SARS-CoV-2 positivity results between the first (5%) and second (17%) stages may be explained by the overall trajectory of the pandemic in the United States. The discovery cohort primarily comprises individuals enrolled relatively early in the pandemic, while the replication cohort enrolled at the height of the winter surge. Likewise, the lower rate of asymptomatic infection in the replication cohort (5.8% vs. 14.1% in discovery) may be attributed to many factors related to change over time in the pandemic; such as the prevalence of more virulent viral variants or shifts in public utilization of testing. We also observed significantly older age in asymptomatic individuals compared to those who developed symptomatic infection. This difference may be at least in part explained by subjects’ self-reported reasons for seeking testing for the virus. Perhaps unsurprisingly, the most common reason patients reporting symptomatic infection sought testing was because they experienced symptoms consistent with COVID-19. In contrast, asymptomatic subjects often were tested for the virus in conjunction with their employment status, such as healthcare workers, who may skew older in our study population. While we cannot rule out a biological basis for the age association with asymptomatic infection, we feel that some ascertainment bias may also explain this observation. Regardless, our observations with respect to *HLA* remain highly significant upon adjusting for age.

In summary, we have demonstrated a strong and significant association of a common *HLA* class I allele, HLA-B*15:01, with asymptomatic infection with SARS-CoV-2. Our results have important implications for understanding early infection and the mechanism underlying early viral clearance and may lay the groundwork for refinement of vaccine development and therapeutic options in early disease.

## Supplementary Material

Supplement 1

## Figures and Tables

**Table 1.** Study population demographics

Discovery					
Total	Symptomatic	Asymptomatic			
640	550	90			
	85.9%	14.1%			
	Male	Female		Age (median)	
All	0.20	0.80	NS	38	***p*<0.001**
Symptomatic	0.19	0.81		**37**	
Asymptomatic	0.28	0.72		**44**	
Replication					
Total	Symptomatic	Asymptomatic			
788	742	46			
	94.2%	5.8%			
	Male	Female		Age (median)	
All	0.17	0.83	NS	32	***p*<0.001**
Symptomatic	0.17	0.83		**31**	
Asymptomatic	0.24	0.76		**38**	
Combined					
Total	Symptomatic	Asymptomatic			
1428	1292	136			
	90.5%	9.5%			
	Male	Female		Age (median)	
All	0.19	0.81	***p*=0.02**	34	***p*<0.001**
Symptomatic	**0.18**	**0.82**		**33**	
Asymptomatic	**0.26**	**0.74**		**41**	

NS = non-significant; *p* = p-value

**Table 2.** HLA-B*15:01 is associated with SARS-CoV-2 asymptomatic infection

Asymptomatic Symptomatic						
Discovery	*f*	*f*	OR	95% CI	*p*	*p* ^corr^
HLA-B*15:01	0.1111	0.0473	2.52	1.39–4.42	0.00056	0.017
HLA-B*15:01 (adjusted)			2.45	1.38–4.24	0.0016	0.048
Replication						
HLA-B*15:01 (adjusted)	0.1087	0.0512	2.32	1.10–4.43	0.017	
Combined						
HLA-B*15:01 (adjusted)	0.1103	0.0495	2.40	1.54–3.64	5.67 ×10^−5^	
HLA-B*15:01/15:01 (adjusted)	0.0220	0.0050	8.58	1.74–34.43	0.001	

Adjusted = adjusted for age and sex; *f* = frequency; OR = odds ratio; CI = confidence interval; *p* = p-value; *p*^corr^ = p-value after Bonferroni correction
